# The internal nasal valve: a validated grading system and operative guide

**DOI:** 10.1007/s00405-018-5142-x

**Published:** 2018-10-06

**Authors:** B. Patel, J. S. Virk, P. S. Randhawa, P. J. Andrews

**Affiliations:** Rhinology Department, Royal National, Throat, Nose and Ear Hospital, Gray’s Inn Road, London, WC1X 8DA UK

**Keywords:** Nasal obstruction, Septoplasty, Septorhinoplasty, Nasal inspiratory peak flow, Valve

## Abstract

**Purpose:**

Nasal obstruction is a highly subjective and commonly reported symptom. The internal nasal valve (INV) is the rate limiting step to nasal airflow. A static INV grading score was devised with regard to visibility of the middle turbinate.

**Methods:**

A prospective study of all patients who underwent primary external functional septorhinoplasty in 2017 for nasal obstruction. All patients’ INV score was assessed pre- and postoperatively in a blinded and independent fashion by surgeons of varying seniority.

**Results:**

Twenty-eight patients were studied, with mean age 30.9 years and follow-up 18.8 weeks. Inter-rater and test–retest reliability of INV grading were excellent, with Cronbach’s alpha 0.936 and 0.920, respectively. There was also statistically significant improvement in both subjective and objective postoperative outcome measures including nasal inspiratory peak flows.

**Conclusions:**

We demonstrate a novel, easy to interpret, clinically valuable grading system of the static internal nasal valve that is reliable and reproducible.

## Introduction

Nasal obstruction is a common and highly subjective complaint, but examination findings do not always correlate with patients’ symptoms [1]. There have been numerous attempts to validate clinical, instrumental and qualitative questionnaires to quantify degrees of nasal obstruction with varying successes [2–5].

The American Academy of Otolaryngology clinical consensus statement stated that the internal nasal valve plays a distinct role in nasal obstruction separate from other anatomical pathologies, such as allergy. Furthermore, there was agreement that surgery is an effective treatment option for such cases [6].

Structural nasal obstruction can be caused by a deviated nasal septum (DNS), internal nasal valve (INV) obstruction or external nasal valve (ENV) obstruction. Grading systems are in place for a DNS and ENV collapse but not INV obstruction [7]. Internal nasal valve obstruction can be caused by a static structural abnormality (high septal deviation or an enlarged turbinate) or by a dynamic collapse abnormality of the upper lateral cartilage/lateral nasal wall on inspiration secondary to a weakness in the integrity of the upper lateral cartilage/nasal side wall. Static and dynamic INV collapses are distinct entities but can also coexist.

The internal nasal valve (INV) is located approximately 1.3 cm from the nares and is typically the narrowest portion of the nasal cavity. It is a cross-sectional area bounded medially by the dorsal septum, laterally by the caudal portion of the upper lateral cartilage and inferiorly by the head of the inferior turbinate [4]. The average angle of the INV in a Caucasian ranges from 9° to 15° and inter-racial variance is well recognised, in part due to the size of the inferior turbinate. Collapse of the valve is thought to obey Bernoulli’s principle and as such, is a common cause for nasal obstruction [5].

We have devised a method of analysing the static component of the internal nasal valve by measuring the degree of middle turbinate visualisation, which can serve as a marker of internal nasal valve obstruction. The primary objective of this study is to investigate and assess the validity of our grading system and secondary endpoints are to evaluate its correlation with objective and subjective rhinological outcome measures both pre- and postoperatively.

## Materials and methods

### Patients

A prospective study of all patients who underwent primary external functional septorhinoplasty under the care of both senior authors at the Royal National Throat Nose and Ear Hospital in 2017 for nasal obstruction. Exclusion criteria were patients under 16 years old, inability to give informed consent, incomplete data and those undergoing concomitant procedures.

### Questionnaires, INV grading and NIPF measurements

Pre- and postoperatively all patients completed the Nasal Obstruction Symptom Evaluation (NOSE) score and Sino-nasal Outcome Tool (SNOT-23) questionnaires alongside a Visual Analogue Scale (VAS) comprising a 10-cm linear scale in which patients rated their nasal obstruction (unilateral and bilateral). The SNOT-23 and NOSE scores were chosen as both are established scoring systems in pre- and post-operative evaluation of surgical patients [8].

**Fig. 1:**
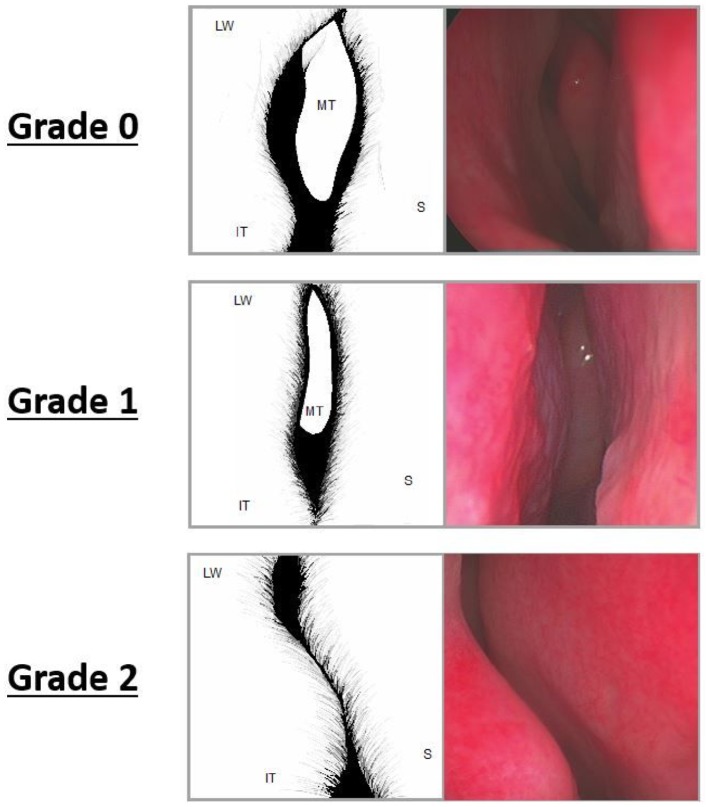
INV grading system. *MT* Middle turbinate, *IT* inferior turbinate, *S* septum, *LW* lateral wall. Measurement is made using a Thudicum’s speculum on anterior rhinoscopy or, ideally, a 0° Hopkins rod placed at the level of the head of the inferior turbinate. Grade 0 the middle turbinate is easily visible including the head. Grade 1 the middle turbinate is partially obscured and in Grade 2 the middle turbinate is not visible

The internal nasal valve (INV) is graded according to the degree of middle turbinate visualisation. On endoscopic imaging, this is assessed in each nostril at rest, at the level of the head of the inferior turbinate. On anterior rhinoscopy INV is graded based on a horizontal line at the level of the head of the inferior turbinate. Grade 0 signifies that the head of the middle turbinate is easily visible. Grade 1 signifies that the middle turbinate is partially obscured. Grade 2 signifies that the middle turbinate is not visible. A maximum grade 2 is given for each nostril (Fig. 1). INV grading was documented on the day of surgery and at the patient’s second postoperative visit. Endoscopic images were taken and this grading was compared between three authors independently and repeated at 3-month post-surgery by these three authors. The assessments were blinded and independent, with each of the authors of differing experience (non-specialist junior doctor, otolaryngology trainee and rhinology consultant).

All subjects were decongested pre and post measurements with phenylephrine (5% lignocaine, 0.5% phenylephrine) to exclude significant mucosal disease and the variability incurred secondary to the nasal cycle. Unilateral and bilateral nasal inspiratory peak flows (NIPF) were taken on the same days as the grading of the INV, with the best result of three attempts used for analysis [9]. This was performed with a peak flow meter attached to a face mask secured over the patient’s nose and mouth. For bilateral measurements, the patient was asked to breathe in through the nose as hard and as fast as possible. For unilateral measurements, one nostril was closed off with tape and the same instructions were followed.

### Operative technique

All patients underwent surgery under the care of two senior authors (PSR, PA). Endoscopic assessment of the INV was performed preoperatively as outlined above. As septoplasty alone around the INV area carries a significant risk of saddle collapse with subsequent reduction in nasal airway, all patients underwent septorhinoplasty via external approach with corrective surgery guided by INV grade. All patients underwent septal corrective surgery with columellar struts placed to reinforce tip support. In the majority of patients, the INV was not adequately corrected with septoplasty and columellar struts alone, therefore spreader grafts were also inserted with cartilage harvested from the septum.

### Statistical analysis

GraphPad^®^ Prism (La Jolla, CA, USA) with paired *t* tests and rank correlation coefficients. Internal consistency was measured with Cronbach’s alpha. A P value less than 0.05 was considered statistically significant. Correlation coefficient, *r*, is considered strongly positive if above 0.5, moderate if above 0.3 and weakly associated if above 0.2. Cronbach’s alpha is considered excellent if more than or equal to 0.9, good if between 0.8 and 0.9 and acceptable if between 0.7 and 0.8.

### Ethical consideration

There was no deviation in our standard of care for any of the patients included in the study and so ethical approval from the hospital board was not required. Informed consent was taken from all patients and data anonymised.

## Results

### Patient demographics

Twenty-eight patients were followed up with 18 male (64.3%), 10 female (35.7%) and a mean age of 30.9 years (95% CI 27.0–34.8). All patients underwent primary external functional septorhinoplasty under the care of the senior authors (PA, PSR). Mean follow-up was 18.8 weeks (95% CI 14.8–22.7). A large proportion of patients (75) were excluded due to incomplete datasets and loss to follow-up.

### Inter-rater reliability and test–retest reliability of static INV grading

Inter-rater reliability (*n* = 112, 3 raters) was excellent, with Cronbach’s alpha 0.936 (95% CI 0.913–0.954).

Test–retest reliability (*n* = 336, over 3 months time period) was excellent, with Cronbach’s alpha 0.920 (95% CI 0.901–0.935).

### Pre- and postoperative outcome measures

Table 1 summarises pre- and postoperative outcome measures.

**Table 1:** Pre- and postoperative outcome measures

	Preoperative mean	Postoperative mean	*p* value
SNOT 23	55.3 (44.6–66.1)	39.6 (29.4–49.8)	0.0012*
NOSE	14.0 (12.2–15.6)	7.5 (5.9–9.0)	< 0.0001*
Right NIPF	63.9 (55.5–72.3)	78.2 (70.0–86.4)	< 0.0001*
Left NIPF	58.4 (50.2–66.6)	76.3 (69.6–83.0)	< 0.0001*
Bilateral NIPF	79.8 (69.9–89.8)	101.4 (92.1–110.8)	< 0.0001*
Right VAS	6.8 (5.8–7.7)	3.9 (3.2–4.6)	< 0.0001*
Left VAS	7.1 (6.1–8.1)	3.6 (2.9–4.4)	< 0.0001*
Bilateral VAS	8.4 (7.8–8.9)	3.8 (3.1–4.5)	< 0.0001*
Right INV grade	1.11 (0.84–1.37)	0.36 (0.12–0.60)	0.0003*
Left INV grade	1.11 (0.80–1.41)	0.32 (0.14–0.51)	0.0001*

Parentheses demonstrate 95% CI confidence interval. NIPF measured in l/min

*Significant *p* value

**Fig. 2:**
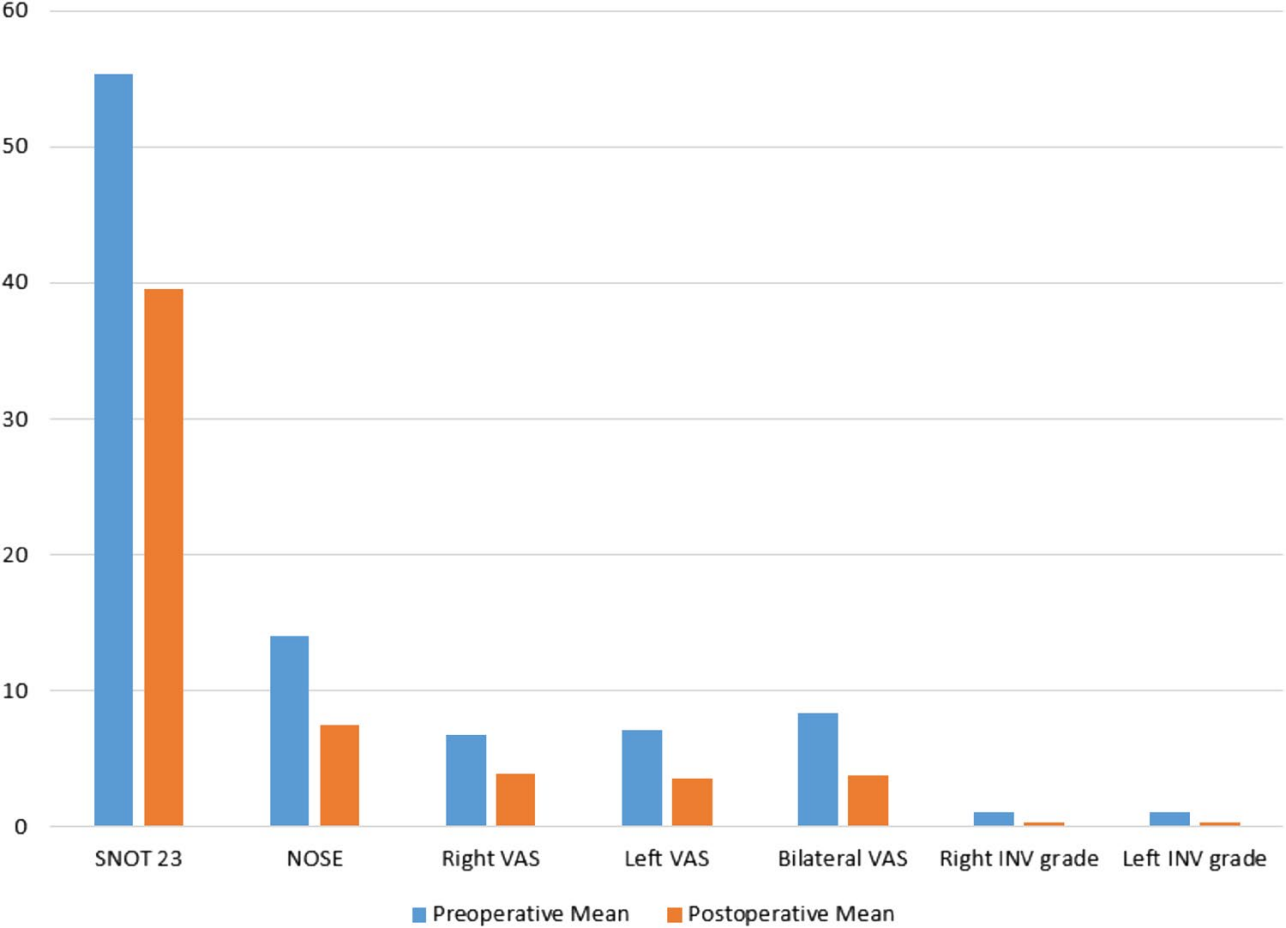
Pre- and postoperative outcome measures

**Fig. 3:**
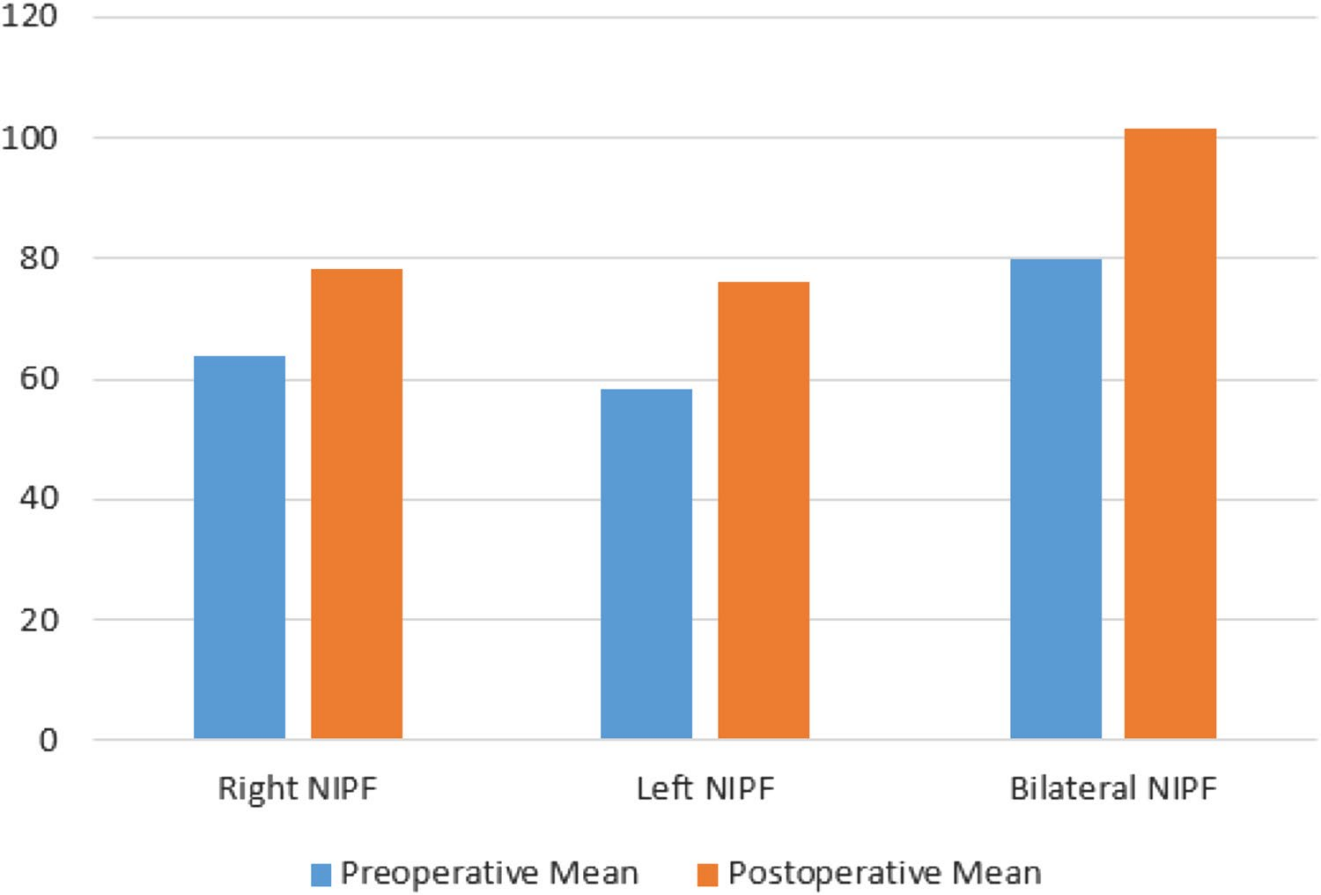
Pre- and postoperative changes in unilateral and bilateral nasal inspiratory peak flow (NIPF, l/min)

This demonstrates statistically significant reductions in subjective scores (SNOT, NOSE, VAS) postoperatively. There was statistically significant improvement in unilateral and bilateral NIPFs postoperatively. In addition, the internal valve grading was significantly reduced postoperatively (Figs. 2, 3).

### Comparative analysis

To explore the relationship between these subjective and objective scoring systems, correlation analysis was performed. This is summarised in Table 2. The main findings include strong positive correlation between NOSE and bilateral VAS scores, moderate positive correlation between SNOT and NOSE scores alongside with a moderate negative correlation between bilateral VAS and bilateral NIPFs and unilateral NIPFs with unilateral VAS. Finally, the INV grading system showed moderate correlation with bilateral VAS scores, NOSE scores, unilateral NIPFs and weak correlation with SNOT-23.

**Table 2:** Selected correlative statistics

Correlation	*r*	*p*
NOSE score vs bilateral VAS	0.54	0.00002*
SNOT vs bilateral VAS	0.21	0.129
SNOT vs NOSE	0.40	0.003*
SNOT vs bilateral NIPFs	− 0.17	0.087
NOSE vs bilateral NIPFs	− 0.23	0.217
VAS bilateral vs bilateral NIPFs	− 0.38	0.004*
Unilateral NIPFs vs unilateral VAS	− 0.45	< 0.0001*
Unilateral NIPFs vs unilateral INV grade	0.30	0.0014*
INV grading vs NOSE	0.32	0.0006*
INV grade vs bilateral VAS	0.41	< 0.0001*
INV grade vs SNOT	0.21	0.0259*

*Significant *p* value

## Discussion

### Synopsis

Grading systems to clinically evaluate nasal obstruction are myriad but lack any consensus or standardisation. In addition, some of these demonstrate no correlation to nasal airflow as demonstrated by Camacho et al. who compared NOSE, VAS and inferior turbinate size [1]. Visualisation of the anatomical INV boundaries remains the key to evaluate these patients.

Our internal nasal valve grading system is a simple and reproducible grading system to objectively assess nasal obstruction. This study demonstrates excellent inter-rater and test–retest reliability (across a large number of observations), which is fundamental to the use of any grading system. This scoring tool is of value for the static component of INV dysfunction. These grades can therefore be affected by a multitude of pathologies such as septal deviation, turbinate hypertrophy, inferior displacement of the upper lateral cartilages or a narrowed pyriform aperture. Clinical acumen is still required to decide how best to achieve expansion of the nasal airway to improve the patient’s symptoms. In addition, the dynamic component of the INV must also be assessed preoperatively. In this study, we selected only primary surgical candidates with no evidence of dynamic collapse. We also ensured that there was little reversibility in nasal airflows post-decongestion to exclude or minimise the impact of mucosal disease. This grading system also serves as an operative guide, in that, we aim for a grade 0 view at the end of the surgery, and thus, for example, in addition to realigning the septum, spreader graft insertion may be necessary to ensure an optimal view of middle turbinate and hence optimise nasal airflow.

This study also demonstrates significant improvements in INV grading postoperatively alongside with other subjective and objective outcome measures. These data will be useful to highlight the efficacy of septorhinoplasty surgery, particularly in view of increasing commissioning restrictions.

In this series, visual analogue scores appear to correlate with unilateral NIPFs. Visual analogue score is often thought to represent the best outcome measure for identifying nasal obstruction [10]. This aids in the validation of unilateral NIPF as an essential routine measure and may represent the best objective marker of nasal obstruction. The moderate correlation between the INV grading system and unilateral NIPF demonstrates the benefits of the grading system and justifies its use as a standard of care.

A previous study in our centre demonstrated postoperative improvement in NIPFs following septorhinoplasty although there was no significant correlation with SNOT scores [4]. In this study, we have shown that unilateral NIPFs do correspond with subjective unilateral and bilateral blockage. This study suggests that VAS, NIPFs and INV grading are the most useful markers. Unsurprisingly, there was good correlation between NOSE and VAS scores. There was no significant correlation with SNOT-23, most likely in view of its wider range of questions rather than focusing on nasal blockage symptoms.

Prospective data collection in the form of questionnaires, grading scales and objective data in the form of NIPFs are valuable at monitoring both medical and surgical interventions and serve as useful tools to monitor outcomes and identify trends. Interestingly, our study found that following surgery there was an improvement in all of the outcomes recorded. It is also beneficial for patients to see how their scores have improved following intervention. Ideally a consensus should be reached for a minimum dataset, much like thyroid surgery, to be recorded by all surgeons to allow comparison of outcomes. This concept was particularly borne out following a recent questionnaire evaluating current ENT practice in measuring nasal obstruction [11].

### Strengths and limitations of the study

We have validated an objective, reliable and reproducible grading system for the static internal nasal valve. This is of clinical value in assessing patients appropriately with nasal obstruction. We have also shown that the INV can be surgically improved with resultant improvements in both subjective and objective outcome measures.

An additional strength of this grading system is its ability to augment the training of septorhinoplasty surgery amongst our juniors. It came to light that by instilling the concept of being able to visualise the middle turbinate bilaterally as an end marker of operative success, juniors found this grading system very valuable, although it is important to be aware of and assess for the role of mucosal disease in these patients alongside with the dynamic aspect of INV dysfunction.

The main limitation of this study is the reduced number of participants due to incomplete datasets and the subsequent limited power of the study. However, within our NHS limitations and given that, at least 112 INV gradings were made by three different observers of varying grades; there were sufficient data to ascertain our primary endpoint, the reliability and reproducibility of the grading system.

A further limitation of this study was that we did not incorporate an additional dynamic component to the grading system which would have evaluated internal valve collapse on deep inspiration. We decided against this as it proved challenging to measure with regards to explaining to the patient the force of deep inspiration required and also we were very conscious to keep this simple and easy to use in our busy clinical setting. In addition, controversy exists regarding the dynamic evaluation of nasal obstruction [3–5]. While acoustic rhinometry may provide a static measure of the cross-sectional area across the INV, the placement of the probe itself splints the nasal airway and therefore invalidates measurement of the INV through this modality. By contrast, a recent study by Pendolino et al., has demonstrated reasonable correlation between unilateral NIPF and unilateral AAR—the gold standard in the measurement of nasal airway resistance [12]. We therefore feel that unilateral NIPF represents a good measurement of nasal airflow.

Furthermore, recent publications question the concept of the INV angle and computational dynamics indicate that the shape of this region is variable, suggesting that assessment with anterior rhinoscopy or endoscopes is not sufficient [2].

## Conclusion

We present an easy to use, reliable and reproducible internal nasal valve grading score that has been validated. We would advocate using this alongside with a combination of subjective and objective measures for all patients undergoing nasal surgery.
